# Management of toxicities associated with targeted therapies for HR-positive metastatic breast cancer: a multidisciplinary approach is the key to success

**DOI:** 10.1007/s10549-019-05261-5

**Published:** 2019-05-07

**Authors:** Marina Elena Cazzaniga, Romano Danesi, Corrado Girmenia, Pietro Invernizzi, Alessandra Elvevi, Massimo Uguccioni, NetworkER+, Laura Amaducci, Francesco Atzori, Livio Blasi, Chiara Butti, Elena Collovà, Enrico De Conciliis, Alessandra Fabi, Antonio Febbraro, Ornella Garrone, Lorenzo Gianni, Francesco Giotta, Nicla La Verde, Andrea Michelotti, Raffaella Palumbo, Ida Paris, Mirco Pistelli, Laura Pizzuti, Daniela Rubino, Maria Rosaria Valerio, Fable Zustovich

**Affiliations:** 1 Department of Medical Oncology & Phase 1 Research Centre ASST-Monza, Via Pergolesi 33, 20900 Monza, Italy; 2 Unit of Clinical Pharmacology and Pharmacogenetics, Department of Clinical and Experimental Medicine, University of Pisa, Pisa, Italy; 3 Department of Hematology, Oncology and Dermatology, Azienda Policlinico Umberto I, Sapienza University, Rome, Italy; 4 Division of Gastroenterology and Center for Autoimmune Liver Diseases, Department of Medicine and Surgery, University of Milan Bicocca, Monza, Italy; 5 Cardiology1/CCU, San Camillo Hospital, Rome, Italy

**Keywords:** Abemaciclib, Advanced breast cancer, Everolimus, Neutropenia, Palbociclib, Ribociclib

## Abstract

**Purpose:**

Agents targeting HR-positive, HER2-negative locally advanced or metastatic breast cancer have improved patient outcomes compared with conventional single-agent endocrine therapy. Currently, approved targeted agents include everolimus and three CDK4/6 inhibitors, palbociclib, ribociclib, and abemaciclib. Unlike the well-characterized and easily manageable safety profile of endocrine therapies, adverse events associated with targeted therapies are complex and potentially severe. Their prompt recognition and treatment, crucial for prolonged endocrine sensitivity and survival, may be challenging and requires a multidisciplinary effort and a good knowledge of drug interactions.

**Methods:**

We reviewed the current evidence on the drug safety of targeted agents for metastatic breast cancer currently used in clinical practice in Italy, supported by the clinical experience of Italian oncologists with expertise in the field.

**Results:**

All oncologists had used CDK4/6 inhibitors in clinical practice and/or within a clinical trial. The clinical management of toxicities, including dose adjustments, treatment interruptions, and concerns regarding special populations is discussed, and the management of relevant adverse events, related to individual agents and class-specific, toxicities is reviewed. Hematologic toxicities have the greatest impact on clinical management of the disease and on patients. Although toxicities associated with the new treatments result in more visits to the physician and more time and attention with patients, they are manageable, with no need for the oncologist to consult with specialist physicians.

**Conclusions:**

Based on the available evidence and current guidelines, we propose a series of practical recommendations for multidisciplinary clinical management of the various toxicities associated with the addition of targeted agents to endocrine therapy.

## Introduction

The development of targeted agents for the treatment of hormone receptor (HR)-positive, HER2-negative locally advanced or metastatic breast cancer has significantly improved progression-free survival (PFS) and rates of objective response and clinical benefit compared with conventional single-agent endocrine therapy [1–8]. Currently, approved targeted agents include everolimus, an oral inhibitor of the mammalian target of rapamycin (mTOR) [9] and three oral selective inhibitors of cyclin-dependent kinases (CDK) 4 and 6, palbociclib, ribociclib, and abemaciclib [10–12].

Compared with the well-characterized and easily manageable safety profile of endocrine therapies, usually related to symptoms typically associated with estrogen deprivation, including arthralgia, hot flashes, and fatigue, adverse events associated with targeted therapies are distinct and more complex. Results from the registration trials have shown that combining targeted agents with endocrine therapies substantially increases the incidence of grade 3–4 adverse events compared with conventional single-agent endocrine therapy [1, 2, 4, 5, 7, 8]. Everolimus has been associated with stomatitis and non-infectious pneumonitis, while neutropenia is the most common toxicity related to CDK4/6 inhibitors [13–18]. Prompt recognition and management of these toxicities is vital for treatment persistence and, therefore, for maximizing survival. However, this may pose a challenge to many oncologists, as experience in clinical practice with novel treatment strategies for metastatic breast cancer is still limited. In addition, for fear of adverse events, many oncologists may not prescribe targeted therapies to elderly or frail patients, precluding them from effective treatment options [14, 19]. Another issue of importance for optimal use of strategies combining endocrine therapy and targeted agents is knowledge of drug interactions related to everolimus and CDK4/6 inhibitors.

In the present paper, we review the relevant evidence concerning drug interactions and safety profiles of everolimus and the CDK4/6 inhibitors in use in clinical practice in Italy for the treatment of HR-positive metastatic breast cancer and discuss the toxicities associated with this novel approach to the treatment of breast cancer. Based on the available evidence and current guidelines, we propose a series of practical recommendations for clinical management of the various toxicities associated with the addition of targeted agents to endocrine therapy.

## Methods

A multidisciplinary panel of five Italian clinicians, including an oncologist, a pharmacologist, a hematologist, a gastroenterologist, and a cardiologist, convened at a meeting organized within the NetworkER+ project (held in Rome, Italy in January 2018). A group of 20 oncologists from the HERMIONE network also attended the meeting. HERMIONE is a network platform that was launched in February 2017 to promote communication and collaboration among oncology centers and oncologists involved in the treatment of metastatic HR-positive breast cancer in Italy.

The main objectives of the NetworkER+ meeting were to: review current evidence concerning the drug interactions and safety profile of targeted agents used in metastatic breast cancer; produce recommendations for the optimal management of drug interactions and toxicities related to targeted agents; focus on frail patients with metastatic breast cancer, namely the elderly and those with organ dysfunction.

### Clinical experience

The 20 oncologists at the meeting were from Oncology Units treating ≥ 150 breast cancer cases per year, across the entire national territory. All had used CDK4/6 inhibitors in their clinical practice; 60% within a clinical trial. In their experience, hematologic toxicities have the greatest impact on both clinical management of the disease and on patients. The toxicities associated with the new treatments result in more visits to the physician and in more time and attention devoted to patients. However, they are considered to be manageable, with no need for the oncologist to consult with specialist physicians.

### Drug interactions

Drug interactions are an important cause of morbidity. The most relevant pharmacokinetic interactions involve drugs that can inhibit or induce enzymes in the hepatic cytochrome P450 system [16]. Members of the CYP3A family of P450 enzymes are probably the most important of all drug-metabolizing enzymes because of their abundance and ability to process a large number of chemically unrelated drugs from almost every drug class [16]. Strong CYP3A4 enzyme inhibitors in clinical use include the antibiotics clarithromycin, telithromycin, and erythromycin, and the antifungal agents ketoconazole and voriconazole; grapefruit juice is also a strong CYP3A4 inhibitor. Strong CYP3A4 inducers include the antibiotic rifampicin and antiepileptic agents such as barbiturates, phenytoin, and carbamazepine; herbal preparations (St. John’s wort) can also induce CYP3A4. Predicting drug interactions is not easy. A factor that further complicates the understanding of drug interactions is the finding that inhibitors/inducers and substrates of CYP3A4 overlap with those of the drug-transporter protein P-glycoprotein (P-gp) [16]. P-gp acts as an efflux pump-exporting drugs, for example, into the intestinal lumen.

Everolimus and the CDK4/6 inhibitors palbociclib, ribociclib, and abemaciclib are administered orally and have different half-lives, undergo predominantly hepatic metabolism, and are substrates of CYP3A4; everolimus is also a substrate of P-gp (Table 1) [20–23]. The concomitant use of everolimus or a CDK4/6 inhibitor with inhibitors of CYP3A4 (or P-gp for everolimus) may, therefore, result in an increased plasma concentrations and pharmacological effect of the targeted agent, while their concomitant use with an inducer of CYP3A4 (or P-gp for everolimus) may lead to lower plasma concentrations and decreased pharmacological effect. According to the labels of the four targeted agents, the concomitant use of strong CYP3A4 inhibitors or inducers is not recommended (everolimus) or should be avoided (CDK4/6 inhibitors). If unavoidable, the dose of everolimus or CDK4/6 inhibitors should be adjusted as recommended (Table 2). Liver function tests should be performed before initiating treatment with abemaciclib and ribociclib and repeated every 2 weeks for the first two cycles, monthly for the next two cycles, then as clinically indicated [10, 12]. An additional relevant interaction exists for ribociclib with anti-arrhythmic drugs and other medications that prolong the QT interval on the electrocardiogram (ECG). Such medications should be avoided in patients receiving ribociclib. Ribociclib is also a strong to moderate inhibitor of CYP3A4 and should be used with caution with CYP3A4 substrates that have a narrow therapeutic index. With the exception of palbociclib, the targeted agents can be taken regardless of food intake. Palbociclib should be taken with food, as its absorption and drug exposure were shown to be low in the fasted state [11, 24].

**Table 1:** Potential and known pharmacokinetic drug interactions of everolimus and CDK4/6 inhibitors

Targeted agent	Dosage	Mean half-life (h)	Metabolism	Interactions^a^ and recommendations for concomitant use
Everolimus [5, 21, 42]	10 mg, once daily, orally with or without food, in combination with exemestane (25 mg once daily, orally)	≈ 30	• Substrate of CYP3A4 and P-gp • Inhibitor of CYP3A4 and P-gp	• Strong CYP3A4/P-gp inhibitors^b,c^: concomitant use not recommended • Moderate CYP3A4/P-gp inhibitors^c^: use caution and consider dose reduction of everolimus to 5 or 2.5 mg/day; if inhibitor is discontinued, consider a washout period of 2–3 days before returning to original everolimus dosage • Strong/moderate CYP3A4 inducers^d^ should be avoided; if unavoidable, increase daily everolimus dose to 20 mg using ≤ 5 mg-increments applied on days 4 and 8 following start of inducer; if inducer is discontinued, consider 3–5 days of washout, before returning to the original everolimus dose
Palbociclib [11, 24]	125 mg, once daily, orally with food, for 21 days followed by 7 days off treatment, in combination with letrozole (2.5 mg, once daily orally) or fulvestrant (500 mg, intramuscularly, on days 1, 15, 29 and once monthly thereafter)	28.8	• Substrate of CYP3A and SULT2A1 • Weak, time-dependent inhibitor of CYP3A	• Strong CYP3A inhibitors^c^ should be avoided; if unavoidable, reduce palbociclib dose to 75 mg once daily; when inhibitor is discontinued, return to original dose of palbociclib after 3–5 half-lives of the inhibitor • Moderate/weak CYP3A ihibitors^c^: no dose adjustments • Strong CYP3A inducers^d^ should be avoided • Moderate/weak CYP3A inducers^d^: no dose adjustment
Ribociclib [12, 43]	600 mg, once daily, orally with or without food, for 21 days followed by 7 days off treatment, in combination with letrozole (2.5 mg once daily, orally) or another AI	32.0	• CYP3A4 substrate • Strong inhibitor at 600 mg-dose and moderate inhibitor at 400 mg-dose of CYP3A4	• Strong CYP3A4 inhibitors^b^ should be avoided, and alternative medications with less potential to inhibit CYP3A4 should be considered; if unavoidable, dose should be reduced to 400 or 200 mg, once daily; if inhibitor is discontinued, return to original dose of ribociclib after 5 half-lives of the inhibitor • Mild/moderate^c^ CYP3A4 inhibitors: no dose adjustment • Strong CYP3A4 inducers^d^ should be avoided • Moderate CYP3A4 inducers^e^ may lead to decreased exposure, in particular in patients treated with the 400- and 200-mg doses • Caution recommended with CYP3A4 substrates with a narrow therapeutic index^e^ • Anti-arrhythmic drugs and other drugs that may prolong the QT interval^f^ on the ECG: should be avoided
Abemaciclib [10]	• 150 mg twice daily, orally with or without food, in combination with an AI or fulvestrant at their recommended doses • 200 mg twice daily, orally with or without food, as monotherapy	18.3	• CYP3A4 substrate	• Strong CYP3A4 inhibitors^c^ should be avoided; if unavoidable, reduce abemaciclib dose to 100 or 50 mg twice daily (or to 150, 100, and 50 mg twice daily, when used as monotherapy) • Strong CYP3A4 inducers^d^ should be avoided

^a^Potential and/or proven interactions

^b^P-gp inhibitors include, but are not limited to, atorvastatin, clarithromycin, cyclosporine, erythromycin, itraconazole, ketoconazole, quinidine, ritonavir, valspodar, and verapamil

^c^Strong CYP3A4 inhibitors include, but are not limited to, clarithromycin, darunavir, indinavir, itraconazole, ketoconazole, nelfinavir, nefazodone, posaconazole, ritonavir, saquinavir, telithromycin, voriconazole, grapefruit, and grapefruit juice; moderate inhibitors include, but are not limited to, amprenavir, cyclosporine (oral), diltiazem, dronedarone, erythromycin, fosamprenavir, fluconazole, imatinib, and verapamil

^d^CYP3A4 inducers include, but are not limited to, carbamazepine, dexamethasone, efavirenz, enzalutamide, nevirapine, pentobarbital, phenobarbital, phenytoin, rifampin, and St. John’s wort (*Hypericum perforatum*)

^e^CYP3A4 substrates with a narrow therapeutic index include, but are not limited to, alfentanil, cyclosporine, everolimus, fentanyl, sirolimus, and tacrolimus

^f^Drugs that are known to prolong the QT interval on the ECG include, but are not limited to, bepridil, chloroquine, clarithromycin, halofantrine, haloperidol, methadone, moxifloxacin, pimozide, and intravenous ondansetron

*AI* aromatase inhibitor, *CYP* cytochrome P450, *ECG* electrocardiogram, *P*-*gp* P-glycoprotein, *SULT*, sulfotransferase

**Table 2:** Dose modifications recommended for the management of adverse reactions associated with targeted therapies

Agent	Starting dose	First reduction	Second reduction	Third reduction
Everolimus	10 mg once daily	5 mg once daily	Not applicable	Not applicable
Palbociclib	125 mg once daily	100 mg once daily	75 mg once daily	Not applicable
Ribociclib	600 mg once daily	400 mg once daily	200 mg once daily	Not applicable
Abemaciclib				
Combination therapy	150 mg twice daily	100 mg twice daily	50 mg twice daily	Not applicable
Monotherapy	200 mg twice daily	150 mg twice daily	100 mg twice daily	50 mg twice daily

Overall, to minimize the risk of harm due to drug interactions, it is important to be aware of possible interactions. It is imperative to ask patients about the use of other medications, including herbal products. At the same time, patients should be informed about drug interactions and instructed to not take any additional medication (including over-the-counter and herbal products) during treatment with targeted therapies without first consulting with the oncologist. Recommended measures to reduce the risk of adverse effects include using an alternative treatment, adjustment of the dose of targeted agent, and close patient monitoring.

### Safety data from clinical trials

Class-effect toxicities associated with the use of mTOR inhibitors include stomatitis, which encompasses inflammation and ulceration of the oral mucosal lining, non-infectious pneumonitis (a non-malignant inflammatory infiltration of the lung), infections, and metabolic adverse events (hyperglycemia and hyperlipidemia) [13].

According to a recent meta-analysis of six randomized controlled trials (RCTs) investigating CDK4/6 inhibitors in combination with an aromatase inhibitor or fulvestrant in women with advanced breast cancer, the most frequent adverse event (all grades) in the group treated with the combination of CDK4/6 inhibitor plus endocrine therapy was neutropenia (65%) followed by diarrhea (49%), infections (44%), nausea (40%), fatigue (39%), and leukopenia (35%) [25]. Other safety issues reported in clinical trials include hepatobiliary toxicity (ribociclib, abemaciclib), prolongation of the QT interval on ECG (ribociclib), and venous thromboembolism (abemaciclib) [10, 12].

### Stomatitis and pneumonitis

In the BOLERO-2 trial, everolimus-related toxicities included stomatitis, pneumonitis, and hyperglycemia [26]. These toxicities were generally of mild or moderate severity and, with the exception of pneumonitis, occurred early after everolimus initiation (within 8 weeks). Adverse events were generally manageable with dose reduction and interruption for a median duration of 7 days. Of note, appropriate dose reductions for toxicity did not have a negative impact on efficacy [27]. In a meta-analysis of five randomized, double-blind phase three clinical trials of everolimus in patients with solid tumors, including breast cancer, renal cell carcinoma, carcinoid tumors, and pancreatic neuroendocrine tumors, the rate of stomatitis was 67% [16]. Stomatitis was mostly grade 1–2, with grade 3–4 reported in < 10% of patients. Stomatitis did not adversely affect PFS. Of note, prophylactic use of a steroid mouthwash has been shown to substantially reduce the incidence and severity of stomatitis in patients undergoing combination therapy with everolimus and an aromatase inhibitor [28].

### Gastrointestinal toxicity

Cases of severe gastrointestinal (GI) bleeding during targeted therapy with everolimus have been reported [29–31]. In two of these reports, gastric antral vascular ectasia (GAVE) was identified as the likely cause of bleeding [29, 30]; GI bleeding was successfully treated with endoscopic hemostasis using argon plasma coagulation after treatment discontinuation. In another case, GI bleeding was associated with treatment initiation and resolved following discontinuation [31]. Endoscopy revealed that the bleeding was secondary to erosive gastritis, and several endoscopic interventions were needed to achieve hemostasis.

The rapidly proliferating GI epithelium is one of the most vulnerable tissues to the effects of antiproliferative drugs. Indeed, GI events including nausea, vomiting, and diarrhea are shared by most anticancer drugs. A meta-analysis of four studies with CDK4/6 inhibitors (palbociclib, ribociclib) was performed to assess the risk of GI toxicities associated with CDK4/6 inhibitors [32]. Adding CDK4/6 inhibitors to endocrine therapy was found to marginally increase the incidence of any-grade decreased appetite, nausea, vomiting, and diarrhea with no significant increase in the risk of high-grade GI toxicities compared with control. In the MONARCH 2 study, which demonstrated that abemaciclib significantly extends PFS when added to fulvestrant in women with advanced breast cancer whose disease had progressed while on endocrine therapy, the most common adverse events in the abemaciclib–fulvestrant versus fulvestrant alone arms were diarrhea (86.4% vs. 24.7%), neutropenia (46.0% vs. 4.0%), nausea (45.1% vs. 22.9%) and fatigue (39.9% vs. 26.9%) [6]. In most cases, diarrhea was effectively managed using supportive treatment, such as antidiarrheal medications and/or dose adjustments. Diarrhea was also the most frequent adverse event in the MONARCH-3 trial investigating abemaciclib plus a nonsteroidal aromatase inhibitor (NSAI) as initial therapy in postmenopausal women with advanced breast cancer [3]. Diarrhea was reported by 81.3% of patients, but was mostly of grade 1 (44.6%). The most frequent grade 3 or 4 event was neutropenia (21.1% vs. 1.2% in the group with a NSAI alone), diarrhea (9.5% vs. 1.2%), and leukopenia (7.6% vs. 0.6%). Low-grade diarrhea was managed in most cases with conventional antidiarrheal medications and dose adjustment.

### Neutropenia

The frequencies of CDK4/6 inhibitor-related grade 3–4 neutropenia reported in RCTs were highest with palbociclib (54–66%, PALOMA-2 and 3 trials) and ribociclib (27–59%, MONALEESA-2 trial) and lowest with abemaciclib (21–27%, MONARCH-2 and 3) [1–3, 6, 18, 33–35]. According to a detailed safety analysis of the PALOMA-3 trial comparing fulvestrant plus palbociclib versus fulvestrant alone (median follow-up, 8.9 months), neutropenia was the most common grade 3 (55%) and 4 (10%) adverse event in patients receiving combination therapy [18]. However, febrile neutropenia was reported only in 0.9% and 0.6% of patients treated with fulvestrant–palbociclib and fulvestrant alone, respectively. Dose modifications for grade 3–4 neutropenia did not compromise PFS. The consequences of myelosuppression experienced during palbociclib treatment are different from those associated with chemotherapy-induced myeloablation [18]. The neutropenia associated with CDK4/6 inhibitors is effectively and rapidly managed by dose delay, interruption, or reduction and without the use of granulocyte colony-stimulating factors (G-CSF). This suggests that mature white blood cells are present in the bone marrow and can rapidly enter the blood circulation when drug levels decrease [18]. In contrast, chemotherapy results in the destruction of progenitor cells leading to more persistent and severe neutropenia.

### Elevation of liver enzymes

The CDK4/6 inhibitors, ribociclib and abemaciclib, have been associated with hepatobiliary toxicity [3, 4, 34, 35]. In the MONALEESA-2 study, grade 3 or 4 elevations in alanine and aspartate aminotransferase levels were reported in 9.3% and 5.7%, respectively, of patients receiving ribociclib plus letrozole versus letrozole alone and have also been observed with other CDK4/6 inhibitors in combination with aromatase inhibitors. Most cases of liver-enzyme elevation were asymptomatic and reversed by dose adjustment. A detailed analysis of the safety and health-related quality of life from the MONALEESA-2 trial confirmed that hepatobiliary toxicity was more common in patients treated with ribociclib plus letrozole than in those treated with letrozole alone, with the onset of such events more common during the first 12 months of treatment (21.3% vs. 9.5% for ribociclib plus letrozole vs. letrozole alone) [34].

Abemaciclib was also associated with a higher rate of hepatic transaminase elevations in the abemaciclib arm of the MONARCH-3 trial [3]. These were generally manageable with dose reduction or dose omission and were resolved with drug discontinuation.

### QTc prolongation

Some drugs can cause a delay in cardiac repolarization, measured as prolongation of the QT interval on ECG. Delay in cardiac repolarization can increase the risk of cardiac arrhythmias, most notably torsades de pointes [3]. In the MONALEESA-2 trial comparing ribociclib combined with letrozole versus letrozole alone for first-line treatment of postmenopausal women with advanced breast cancer, the most frequent adverse event in the ribociclib arm was neutropenia (74.3% vs. 5.2% in the letrozole alone arm), followed by nausea (51.5% vs. 28.5%), infections (50.3% vs. 42.4%), fatigue (36.5% vs. 30.0%), and diarrhea (35.0% vs. 22.1%) [33]. These events were mostly grade 1 or 2. An increase of more than 60 ms from baseline in the QTcF (QT corrected by Fridericia) interval occurred in nine patients (2.7%) in the ribociclib group and in no patients in the placebo group. In the second interim analysis of the MONALEESA-2 trial (at a median follow-up of 26.4 months), safety results were comparable with those reported in the first analysis [4]. A > 60 ms prolongation from baseline in the QT interval occurred in ten patients (3.0%) in the ribociclib plus letrozole group and one patient (0.3%) in the letrozole group. A total of 12 (3.6%) patients receiving ribociclib plus letrozole had at least one QT of > 480 ms versus two patients (0.6%) in the control arm; in the ribociclib plus letrozole group, 11 of these patients had been reported at the time of the first analysis. Two of the 12 patients in the ribociclib plus letrozole group had received a concomitant prohibited medication with a known risk to prolong QT; three of the 12 patients had dose interruption, but this was due to QT prolongation only in 1 patient. No arrhythmia (torsades de pointes) was reported in the ribociclib group. Together these findings confirm that ECG changes observed with targeted therapies are mostly asymptomatic and that QT prolongations are managed effectively by adjusting the ribociclib dose.

### Clinical management of toxicities

Dose adjustments and/or temporary interruption due to adverse reactions are recommended for all targeted agents based on individual safety and tolerability [9–12]. Table 2 shows the dose modifications recommended in the labels of everolimus, palbociclib, ribociclib, and abemaciclib. Of note, the labels state that if further dose reduction is required below the lowest dose indicated, treatment should be permanently discontinued. Overall, dose adjustment is usually not required for adverse reactions of grade 1, while treatment should be discontinued when the adverse reactions are grade 4. For grade 2 and 3 adverse reactions, temporary treatment interruption should be considered until symptoms improve to grade ≤ 1. When this improvement is achieved, treatment should be resumed at the next lower dose. For example, in patients developing grade 2 everolimus-related non-infectious pneumonitis, everolimus should be interrupted until symptoms improve to grade ≤ 1; treatment should be reinitiated at 5 mg daily. If symptoms fail to improve within 4 weeks, treatment should be permanently discontinued. The same is recommended for grade 3 non-infectious pneumonitis; in this case, if toxicity recurs at grade 3 after resuming treatment at 5 mg daily, everolimus should be permanently discontinued. If patients develop grade 2 stomatitis, everolimus should be interrupted until recovery to grade ≤ 1 and reinitiated at the same dose; if stomatitis recurs at grade 2, treatment should be interrupted until recovery to grade ≤ 1 and reinitiated at 5 mg daily. In the case of grade 3 stomatitis, everolimus should be interrupted until recovery to grade ≤ 1 and resumed at 5 mg daily. The approach to management of other relevant safety issues related to targeted therapies is discussed in detail below.

### Management of neutropenia and prevention of infections

Neutropenia induced by CDK4/6 inhibitors is reversible and can be readily managed by dose interruption or modification without compromising treatment efficacy. The propensity to develop higher-grade neutropenia during treatment with CD4/6 inhibitors can generally be recognized within the first months of treatment [18]. Appropriately tailored dose adjustments should be promptly implemented to reduce the risk of recurrent episodes of severe neutropenia and/or febrile neutropenia [18]. It is, therefore, important to monitor absolute neutrophil counts early during treatment so that timely dose adjustments can be implemented in patients experiencing grade 3–4 neutropenia.

**Fig. 1:**
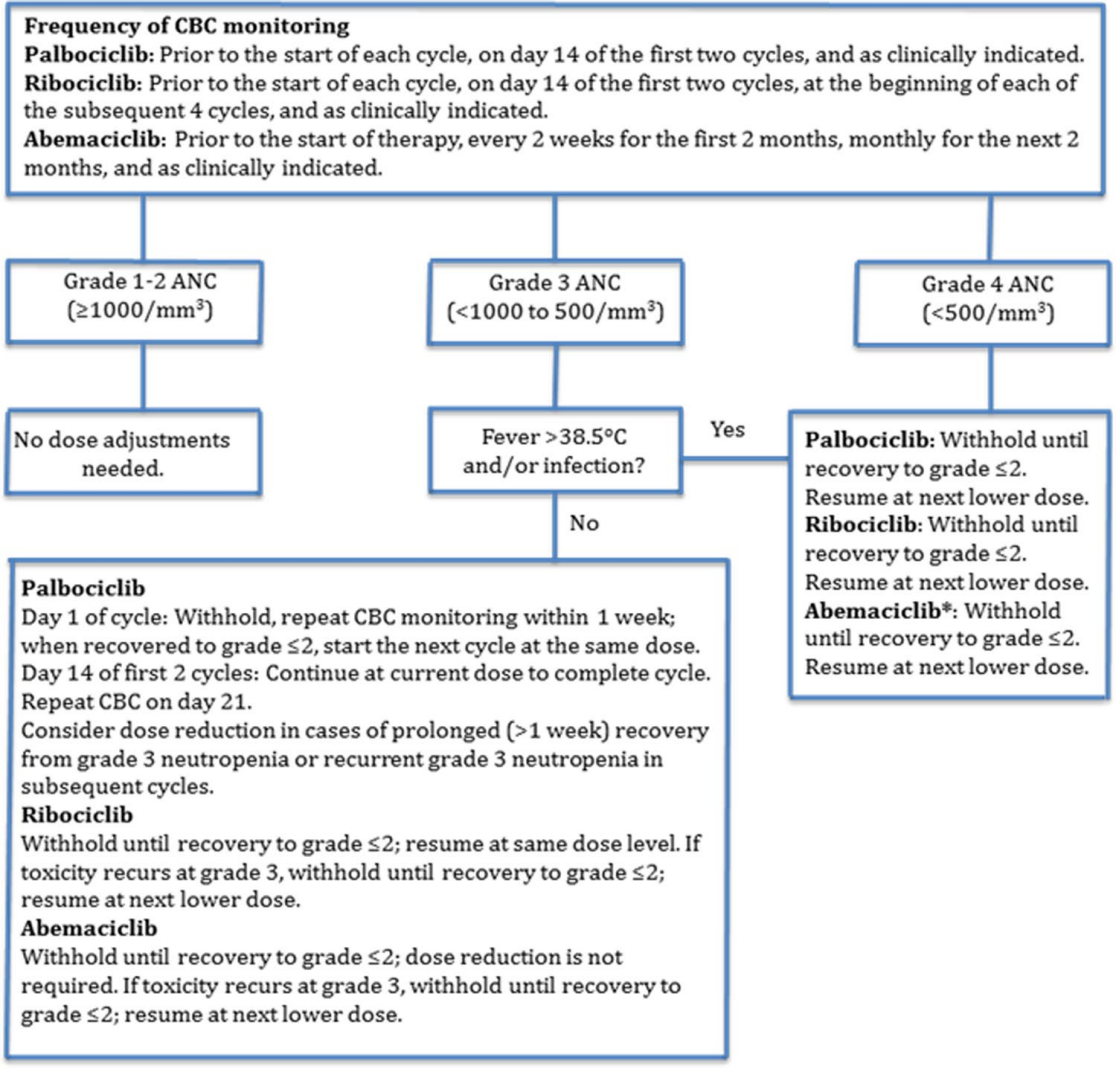
Management of CDK4/6-related neutropenia as recommended in the labels of palbociclib, ribociclib, and abemaciclib. For the recommended dose adjustments, please refer to Table 2. *ANC* absolute neutrophil count, *CBC* complete blood count. *The label of abemaciclib does not differentiate between grade 3 neutropenia with or without fever > 38.5 °C and/or infection. Reproduced with permission from Spring et al. [17]

Figure 1 shows the recommended algorithm for the management of neutropenia associated with palbociclib, ribociclib, and abemaciclib [10–12]. Options for management of neutropenia and infections include dose reduction or drug interruption. Patients who develop grade 2 neutropenia during treatment have an increased risk of developing higher-grade neutropenia and febrile neutropenia. They should, therefore, be closely monitored (complete blood count) throughout treatment with CDK4/6 inhibitors. As for infections secondary to neutropenia, antibacterial prophylaxis should be administered only if grade 4 neutropenia persists for > 7 days and particularly in the presence of mucositis. Vaccination against *Streptococcus pneumoniae* before initiating therapy and annual influenza vaccination is recommended for all patients. The American Society of Clinical Oncology (ASCO) guidelines recommend hepatitis B virus (HBV) screening in all cancer patients treated with conventional chemotherapy or targeted therapies [36]. Based on the serological profile of HBV infection, a different therapeutic strategy will be needed (i.e., treatment or prevention of viral reactivation) [37].

### Management of diarrhea, nausea, and vomiting

In the absence of signs of infection, diarrhea should generally be managed using non-pharmacologic interventions, including hydration, appropriate diet, and avoidance of diarrhea-inducing agents. However, in the case of abemaciclib, the Summary of Product Characteristics directs that treatment with antidiarrheal agents, such as loperamide, should be started at the first sign of loose stools [10]. Recurrent or high-grade diarrhea requires dose reduction. Antidiarrheal medication (loperamide) can also be used. Nausea and vomiting should be treated with antiemetics, including metoclopramide, prochlorperazine, haloperidol, or serotonin-receptor antagonists as needed. Caution should be taken when prescribing symptomatic therapies because of potential drug interactions (Table 1). Particular attention is needed with the concomitant administration of ribociclib with antiemetics (e.g., intravenous ondansetron, dolasetron, metoclopramide, diphenhydramine, haloperidol) because of the risk of QT interval prolongation [38, 39]. With regards to palbociclib, rabeprazole (a proton pump inhibitor) decreases its serum concentration and H2-receptor antagonists or locally acting antacids should be used for the management of nausea. Dexamethasone and aprepitant may, respectively, decrease or increase serum levels of palbociclib; possible alternatives are metoclopramide and domperidone [32].

### QT interval prolongation

**Fig. 2:**
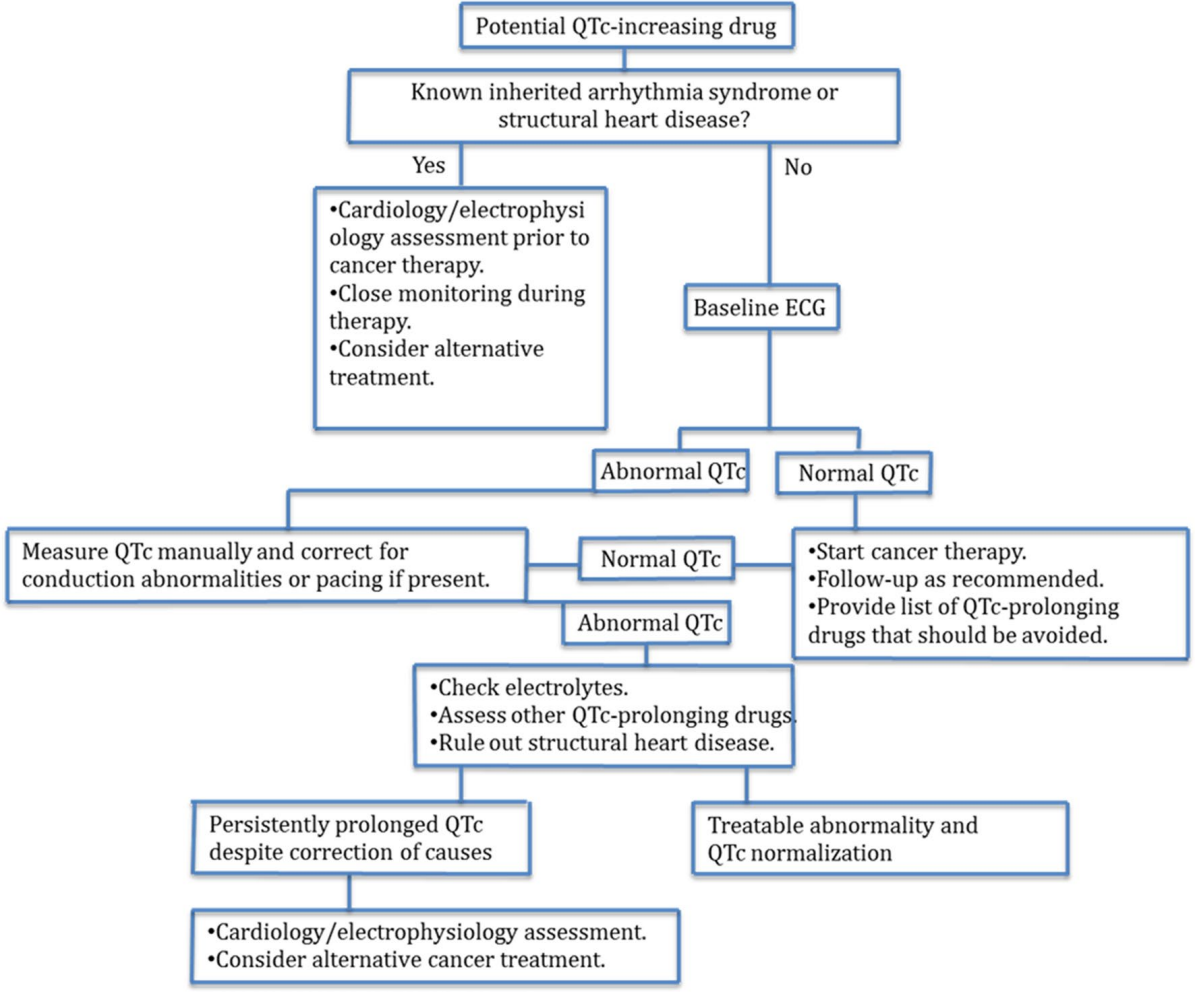
Assessment of patients at risk of QTc prolongation or with QTc prolongation before or during cancer treatment. Reproduced with permission from Porta-Sanchez et al. [40]

Prolongation of the QT interval has been frequently associated with cancer therapies, with or without targeted agents [40]. Substantial prolongation of QT (> 500 ms) is more frequent with targeted therapies [40]. Of note, the incidence of major arrhythmias and myocardial infarction caused by therapy-related QT prolongation is very low [40]. Patients at risk of QT prolongation or with QT prolongation before or during cancer therapy should be assessed as outlined in Fig. 2 [40]. In cancer patients, altered electrolyte levels (hypokalemia, hypocalcemia, and hypomagnesemia) are often due to reduced electrolyte intake, diarrhea, vomiting, fever with sweating, use of laxatives, and therapy with steroids. Furthermore, the use of drugs with potentially synergic effects on QT prolongation (Table 1) and structural cardiomyopathy can also contribute to QT prolongation. A cardiologist should be consulted in the following cases: QT prolongation > 500 ms; prolonged QT during treatment and presence of symptoms of heart disease; history of arrhythmias; history of presyncope or syncope with a likely cardiac origin; prolonged QT and bradycardia < 60 bpm (these patients should undergo ECG monitoring in a cardiology unit).

When initiating treatment with ribociclib, the following points should be kept in mind: (1) QT > 500 ms, severe arrhythmias, or sudden cardiac death related to targeted therapies are very rare; (2) At the first manifestation of QT prolongation, a manual measurement should be performed; (3) If prolonged QT is confirmed, reversible electrolyte alterations should be excluded; (4) Treatment with ribociclib can be initiated in patients with QT < 450 ms; (5) Avoid concomitant administration of drugs that can prolong the QT; (6) ECG should be measured at baseline, 14 days from the beginning of treatment, and at the beginning of the second cycle of treatment; (7) Electrolyte levels should be measured before initiating treatment and if there is an indication for additional monitoring.

### Treatment of elderly and frail patients

A significant proportion of patients with breast cancer are aged ≥ 65 years. Management of toxicities related to targeted therapies may be particularly challenging in this age group because of the presence of comorbidities and frailty. Due to the frequent use of polytherapy by older patients, the management of drug interactions may be particularly complex. Older patients are generally underrepresented in oncology clinical trials, including those that have investigated targeted therapies for HR-positive, HER2-negative metastatic breast cancer [41]. Subgroup analyses and a recent systematic review have shown that adding everolimus or a CDK4/6 inhibitor to endocrine therapy is, however, a feasible strategy in patients aged ≥ 65 years, resulting in improved survival, response rates, and clinical benefit [19, 27, 41]. In these analyses, the safety profile of everolimus and CDK4/6 inhibitors was consistent with that observed for the overall study population [41]. As a consequence, according to the prescribing information, there is no need for dose adjustments of everolimus and CDK4/6 inhibitors in patients aged ≥ 65 years, based exclusively on age [9–12]. Subgroup analyses have also shown that the toxicities induced by targeted therapies in older patients were effectively managed by dose interruptions or reductions, as in the overall population [19].

Based on published evidence, current guidelines, and personal experience, we strongly recommend initiating targeted therapy with CDK4/6 inhibitors in elderly patients at the full licensed dose. Close monitoring to ensure the early detection of adverse events is highly recommended, as toxicities may rapidly worsen in older individuals. Currently, no data are available regarding the use of CDK 4/6 inhibitors in patients with organ dysfunction; these patients should be managed as recommended in the labels of the various targeted agents.

## Conclusions

The addition of targeted agents, everolimus, or CDK4/6 inhibitors to endocrine therapy has considerably improved the outcomes of patients with metastatic HR-positive, HER2-negative breast cancer. This strategy is, however, associated with an increased risk of class-specific toxicities that are potentially serious. Most adverse events associated with targeted therapies have an early onset and can generally be readily managed by dose adjustments and temporary interruptions (Box 1). Early treatment of adverse events is crucial. In clinical practice, the optimal implementation of targeted therapies will require a multidisciplinary effort, the ability to stratify patients according to the risk of adverse events, treatment individualization, and close patient monitoring. Efforts aimed at increasing patient awareness of treatment-related toxicities will also be required.

Box 1 Summary of main recommendations for management of toxicities associated with targeted therapies

- Where possible, strategies to prevent expected adverse events should be considered (e.g., prophylactic use of steroid mouthwashes to prevent stomatitis; vaccinations to prevent common infections).
- Early management of toxicities is very important, and thus the prompt recognition of signs and symptoms is crucial. Patients should be informed about treatment-related toxicities.
- Dose reductions for grade 2–3 adverse events are feasible in many instances, with no detrimental effect on efficacy. Grade 1 toxicities usually do not require dose modifications, while grade 4 toxicities should prompt permanent treatment discontinuation.
- Careful consideration of drug interactions (CYP3A4 inhibitors/inducers, as well as QT-prolonging drugs) is required when prescribing medications for the treatment of adverse events.
- The co-administration of strong inhibitors or inducers of CYP3A4 with everolimus and CDK4/6 inhibitors should be avoided. Alternative medications should be given (e.g., metoclopramide and domperidone as antiemetics; loperamide as antidiarrheal agent).
- Neutropenia induced by CDK4/6 inhibitors is reversible and can be readily managed by dose interruption or modification without compromising treatment efficacy, as described in the drug labels.
- Antidiarrheal agents, such as loperamide, should be started at the first sign of loose stools with abemaciclib; otherwise, diarrhea induced by CDK4/6 inhibitors should initially be treated with non-pharmacologic interventions; antiemetics can be used for nausea and vomiting, after evaluation of possible drug interactions.
- Liver function tests should be performed before initiating treatment with abemaciclib and ribociclib and liver function monitored throughout treatment.
- QT interval prolongation has been associated with targeted therapies; however, major arrhythmias and myocardial infarction have been rarely reported.
- Patients should be seen by a cardiologist when: QT prolongation is > 500 ms; prolonged QT during treatment and presence of symptoms of heart disease; history of arrhythmias; history of presyncope or syncope with a likely cardiac origin; prolonged QT and bradycardia < 60 bpm.
- Older patients (≥ 65 years) can be treated with targeted therapies; the profile of adverse events is similar to that of younger patients. No dose adjustments are required based solely on age.
- Drug interactions need particular consideration in older patients on polytherapy.
